# Consanguinity Associated with Increased Prevalence and Severity of Bipolar Disorder in Pakistan: A Case Report Highlighting the Genetic Link

**DOI:** 10.7759/cureus.1467

**Published:** 2017-07-13

**Authors:** Muhammad Aadil, Aitzaz Munir, Hasnain Arshad, Feham Tariq, Muhammad Jahanzaib Anwar, Noman Amjad, Anum Akhlaq

**Affiliations:** 1 Department of Medicine, FMH College of Medicine; 2 Department of Psychiatry, Howard University Hospital; 3 Department of Internal Medicine, Howard University Hospital; 4 Internal Medicine, Services Hospital; 5 Department of Internal Medicine, Rush University Medical Center; 6 Psychiatry Department, Nishtar Hospital; 7 Department of Medicine, FMH College of Medicine and Dentistry

**Keywords:** bipolar mania, genetics, family health, mania, young age, psychiatry, mini mental state examination, antipsychotic medications

## Abstract

This case report highlights the genetic link associated with bipolar disorder and rising prevalence of such cases in Pakistan due to the lack of knowledge and understanding of the disease. It also shows that a family history of bipolar disorder is associated with more aggressive episodes, early onset, and treatment relapse. Further studies are warranted to fully understand the pathophysiology of genetic linkages causing bipolar disorder so we can understand the natural course of illness and provide effective treatment. We report the case of a 25-year-old girl who presented to the hospital with severe mania and had around 20 episodes of acute mania in the last ten years. Her parents (first cousins) and brother all had a diagnosis of bipolar disorder.

## Introduction

Bipolar disorder (BPD), a manic-depressive recurrent illness entered the DSM (Diagnostic and Statistical Manual Of Mental Disorders) in 1980. It involved only one type at that time, the classic manic illness that needed hospitalization named as bipolar disorder I. The other types that were later included are bipolar disorder II (hypomania and depression), bipolar disorder not otherwise specified, and cyclothymia [1]. In this case report, we highlight the genetic link and severity of BPD and how it should be managed. BPD has a multifactorial etiology. Its development is influenced by genetic as well as environmental factors. Large genome-wide association studies (GWAS), in which genetic risk allelic variants for the disorder could be replicated for the first time, marked the breakthrough in the identification of the responsible risk genes [2]. A replication of the GWAS study done on a large Latino cohort with BPD using family-based approach, where two immediate family members of each patient were included, showed that 32.1% and 17.8% of single nucleotide polymorphism (SNPs) were associated with narrow and broad BPD phenotype respectively.  Two markers in narrow BPD phenotype named rs230529 and rs230535 in NFKB1 retained statistical significance [3].

In the current case, our patient presented with manic episodes at the age of 25, but she had been having episodes of aggression, talkativeness, and over activity since adolescence.The patient had a strong family history as both her father and her brother had bipolar disorder controlled by medication. The early-onset of this disease and family history is usually associated with a worse outcome. The two risk factors contributing to the early onset of bipolar disorder are genomic loading and environmental factors. Being a genetic carrier, she had an early onset and a more severe disease [4].

## Case presentation

A 25-year-old female presented to our facility with aggressive behavior and over activity for the last six weeks. Her mother was the informant and she seemed to be well aware of her disease. She described that her daughter had approximately 18-20 of such episodes for the last ten years and the last episode was about one month back when she developed aggressiveness without any inciting event. She walked out of her home barefoot and called the emergency numbers for complaining against her husband whom she has been abusing for the last couple of hours. During the illness, she visited few physicians and took multiple antipsychotics. She was admitted three times to an inpatient psychiatric unit for the same illness. This time she developed the same aggressive behavior after she had a fight with a shopkeeper and started verbally abusing him. The neighbors and brought her back home. She remained aggressive and abusive at home as well, especially towards her brother and her mother. She also developed a sense of paranoia and suspicion towards her mother and her neighbors. She accused them of bad behavior and thought that they were plotting to send her to a mental institution. According to the mother, the patient had become overactive and roamed around the house without any purpose. She has become talkative and talked continuously about the people she didn’t like and verbally abused them. Her sleep has decreased from eight hours to four hours, and she has not been eating much. Her mother did not describe any history of drug abuse, trauma, suicidal ideation, gait disturbances or febrile illness. Her symptoms had severely affected her daily life and relationships. As per her mother, she has torn all her past medical records.

On reviewing her family history, we found that her father had a similar condition for the past forty years which is now controlled by medications. Both the mother and elder brother have also been diagnosed with BPD and are compliant to treatment. Physical examination of the patient was normal. On mental state examination, the mood is elevated, and there is increase rate, rhythm, the volume of talk and increased flow and flight of ideas preoccupied with gloominess. The patient was admitted to the psychiatric unit and was assessed according to bio-psycho-social model. Her Young Mania Rating Scale (YMRS) was 45; she had a working diagnosis of bipolar type 1 according to the fifth edition of DSM (DSM-5). She was started on olanzapine, sodium valproate, procyclidine, haloperidol, propranolol, and maintained with hydration and nutrition. The patient was hospitalized and was discharged after three weeks with a stable mental condition. She is currently being followed at the outpatient department with no relapse since her last admission.

## Discussion

The current case report highlights a case of severe BPD because of a strong family history. This caused an early onset and more aggressive disease leading to multiple hospitalizations. A differential diagnosis included major depressive disorder, personality disorder, substance abuse/medication-induced bipolar, and attention deficit hyperactivity disorder. A diagnosis of BPD was established after checking laboratory tests, ruling out substance abuse, and confirming all requirements of the diagnostic criteria. The diagnostic criteria for BPD (manic episode) as mentioned in DSM-5 are as follows [5]:

A- A distinct period of abnormally and persistently elevated, expansive, or irritable mood and abnormally and persistently increased activity or energy, lasting at least one week and present most of the day, nearly every day (or any duration if hospitalization is necessary).

B-During the period of mood disturbance and increased energy or activity, three (or more) of the following symptoms (four if the mood is only irritable) are present to a significant degree and represent a noticeable change from usual behavior:

1- inflated self-esteem or grandiosity.

2-Decreased need for sleep (e.g., feels rested after only three hours of sleep).

3-More talkative than usual or pressure to keep talking.

4-Flight of ideas or subjective experience that thoughts are racing.

5-Distractibility (i.e., attention too easily drawn to unimportant or irrelevant external stimuli), as reported or observed.

6-The increase in goal-directed activity (either socially, at work or school, or sexually) or psychomotor agitation (i.e., purposeless non-goal-directed activity).

7-Excessive involvement in activities that have a high potential for painful consequences (e.g., engaging in unrestrained buying sprees, sexual indiscretions, or foolish business investments).

C- The mood disturbance is sufficiently severe to cause marked impairment in social or occupational functioning or to necessitate hospitalization to prevent harm to self or others, or there are psychotic features.

D-The episode is not attributable to the physiological effects of a substance (e.g., a drug of abuse, medication, other treatment) or another medical condition.

 

As mentioned in the national comorbidity survey replication in 2007, the lifetime prevalence for BP-I, BP-II, and subthreshold BPD in the USA is 1.0%, 1.1%, and 2.4% respectively [6]. Though the exact prevalence in Pakistan is not known because of limited research data, a cross-sectional study found it to be alarmingly high, around 14.3% among students, with no significant difference among gender [7]. The extremely high prevalence of bipolar disorder might be linked to increased prevalence of consanguineous marriages in Pakistan. A study conducted in 1998 showed that in urban and rural areas of Pakistan, approximately 60% of marriages were consanguineous and of this 80% of them were first cousins [8]. The finding is quite similar to a study done in the Northeastern part of the Nile delta region of Egypt which also showed an increased prevalence of BPD-1 associated with consanguinity (of all the cases of BP-1, 36.5% were with consanguineous parents, and of all controls, 17.7% were with consanguineous parents P=0.004) [8]. The prevalence might be even higher in Pakistan because of limited psychiatric health care facilities and underdiagnosis of BPD.  BPD is differentiated from major depressive illness by mania or hypomania symptoms, as depression is present in both. Such patients also go undiagnosed as they are often suspected of substance abuse or some sort of a personality disorder.

Bipolar disorder is a very disabling lifelong condition. Only a few people in Pakistan seek medical treatment because of the stigma related to mental illness. There is a genetic link involved in its increased prevalence and more studies on pharmacogenetics are required to target the problem. Another study has proven that the brains of people with BPD had upregulated mRNA expression of the choline dehydrogenase gene (CHDH), located on chromosome 3p21.1, as compared to that of healthy individuals. It has been identified that a single nucleotide polymorphism named rs983659 showed a consistent association with BPD (P meta = 5.72 × 10­4) [3]. In addition to these, rare variants with a higher penetrance are expected to play a role in disease development. Current treatment guidelines are focused on the phase of BPD which includes acute mania, mixed episodes and bipolar depression, and prophylaxis of such episodes. The FDA approved drug therapies for the above-mentioned phases include mood stabilizers which are lithium, lamotrigine, valproate, carbamazepine and atypical antipsychotics. The only medication approved by the FDA for the treatment of acute bipolar depression is olanzapine/fluoxetine combination (OFC) [9]. There is a significant role of psychological treatment in bipolar depression in addition to the drug treatment. Psychoeducation improves adherence to medication and, by recognizing its prodromal symptoms, the patient seeks early treatment thereby decreasing the rate of relapse. Reducing the stressors of life and improving family environment has proven to be a significant factor in preventing relapse.

Our patient was at risk due to a family history; such children should be put on screening and treated appropriately if they have any prodromal symptoms. Omega-3-fatty acids and N-acetyl-cysteine are effective primary interventions for such children. A detailed family history should be carried out in all patient which help us diagnose this condition earlier. Awareness campaigns about diseases linked to cousin marriage and counseling should be provided to counter the burden of this disease in Pakistan.

## Conclusions

We emphasize the need for more studies to understand the genetic involvement of BPD and the pharmacogenetics. Awareness and counseling should be provided to overcome disease burden in society because of consanguinity. This way, we can diagnose and treat the condition earlier and will be able to overcome morbidity associated with this disease.
